# Does Cognitive Behavior Therapy Change Socially Anxious Adolescents’ Behavior during a Public Speaking Task?

**DOI:** 10.1007/s10802-025-01420-z

**Published:** 2026-01-26

**Authors:** Sara L. M. Velthuizen, Esther van den Bos, Anne C. Miers, Jiemiao Chen, P. Michiel Westenberg

**Affiliations:** Faculty of Social Sciences, Leiden University, Wassenaarseweg 52, South Holland Leiden, 2333 The Netherlands

**Keywords:** Social anxiety disorder, Adolescents, Public speaking, CBT, Eye-tracking, Speech disruptions

## Abstract

Public speaking is one of the most commonly feared situations by socially anxious adolescents, often prompting behavioral anxiety markers including gaze avoidance and speech disruptions. While the potential adverse social consequences of behavioral anxiety markers in public speaking contexts have been established, research into how these markers might alter through cognitive behavior therapy is still in its infancy. In this preliminary study, we investigated changes in gaze behavior and speech disruptions from before to after 12 weeks of disorder-specific group cognitive behavior therapy among 41 adolescents aged 11–17 years (*M* = 14.46, 48.78% girls) with social anxiety disorder. Participants spoke for five minutes in front of a pre-recorded classroom audience while wearing an eye-tracker, before and after the *Skills for Academic and Social Success* program. Following treatment, we found an increase in frequency of gaze towards the faces of the audience while speaking, with greater changes among older participants. There were no changes in speech disruptions at the group level. We conclude that therapy may have a positive effect on gaze behavior, and discuss the clinical implications and opportunities for future research in this emerging field of study.

## Introduction

Social anxiety disorder (SAD) is the highly debilitating and persistent fear of being scrutinized by others, particularly during social interactions. This leads to suffering during and avoidance of fear-inducing social situations. The disorder typically has its onset in adolescence and, in youth, the fear is also present in the company of peers (American Psychiatric Association, 2013). SAD is thought to develop at the high end of a continuum, stemming from a combination of genetic and environmental influences, including parental and peer interactions and negative life events (Rapee & Spence, 2004). Cognitive distortions and safety behaviors resulting in poor social performance are involved in the maintenance of SAD (Hodson et al., 2008). Studies have shown that poor social skills can lead to detrimental social outcomes including disinterest in the short term, and lower acceptance and exclusion by peers in the long term (Miers & Masia Warner, 2023).

Yet, very little research has investigated the effect of treatment on behavioral markers of anxiety in a socially anxious population. Instead, treatment effectiveness is often regarded through the lens of remission rates or general symptom-reduction. While this remains the core purpose of treatment, insight into specific behavioral changes may further clarify the extent of remission and inform long-term prognoses. To fill this gap, the aim of this study was to investigate potential changes in socially anxious adolescents’ behaviors during public speaking before and after cognitive behavior therapy (CBT). A public speaking situation was employed because it is one of the most commonly feared situations by socially anxious adolescents (Rao et al., 2007) and lends itself to the study of behavioral markers of anxiety. Moreover, public speaking tasks have been shown to elicit stress responses in clinical and non-clinical populations of adolescents with social anxiety (Asbrand et al., 2019; Inderbitzen-Nolan et al., 2007; Miers et al., 2011). Public speaking tasks have also been shown to trigger negative post-event processing in the days following the task (Asbrand et al., 2019), which according to theory can contribute to the maintenance of the disorder (Clark & Wells, 1995).

The Clark and Wells (1995) model of the development and maintenance of SAD in adults describes how self-focused attention causes socially anxious individuals to base their self-evaluations on negative assumptions about how others view them. This leads to negative expectations, causing them to use overt and covert safety behaviors. An overt safety behavior would be to avoid social situations in general (Hodson et al., 2008) whereas covert safety behaviors include avoidance of eye contact, decreasing the tone of the voice, speaking less, and standing on the outskirts of a group (Rapee & Heimberg, 1997; Wells et al., 1995). This theoretical model is applicable to socially anxious adolescents too (Hodson et al., 2008). Socially anxious adolescents may use these behaviors with the implicit aim of reducing the chance of negative feedback, yet their very presence elicits negative feedback or exclusion from the group, resulting in a self-fulfilling prophecy (Leigh et al., 2021; Leigh & Clark, 2018). Thus, the disorder may in turn worsen and the situation deteriorates further. Reversing this negative spiral through treatment would be highly beneficial (Leigh et al., 2021).

Most studies on safety behaviors have used self-report measures (Piccirillo et al., 2016). Indeed, not all safety behaviors are observable (e.g., rehearsing what to say before giving a speech). However, reliance on the participants’ awareness and relayed impression of their anxiety behaviors are limitations of self-report. Therefore, we concentrate on two of the most commonly studied observable behaviors in SAD during a behavior assessment task: eye-gaze avoidance and restricted speech. Although there is theoretical support for CBT improving behavioral anxiety markers, there is very little empirical evidence. Because of the scarcity of prior research, in particular with adolescent samples, we will discuss both adolescent and adult studies below.

### Behavioral Anxiety Markers During Public Speaking

#### Eye Gaze

Several public speaking studies using eye-tracking technology have provided evidence that adults with SAD look less at the faces of an audience, compared to non-anxious controls (Chen et al., 2015, 2016; Kim et al., 2018). Chen and colleagues (2021) administered a public speaking task, which also included a viewing task, to a community sample of young adults. Participants gave a one-minute speech in front of a pre-recorded audience and were subsequently instructed to perform a viewing task in which they simply looked at the audience for the same duration of time. Participants with high levels of social anxiety showed avoidance of the audience’s faces during public speaking but not while only looking at the audience in the viewing task. The authors concluded that avoidance is therefore not a default response, but rather occurs only when there is risk of negative judgement (see also Alden & Taylor, 2004). On the other hand, adults with SAD have been reported to look less at pictures of faces even when there was no risk of being negatively evaluated (for review see Chen et al., 2020). These two findings together suggest that avoiding faces may generalize to non-evaluative situations over time and with increasing symptoms, thereby becoming a habit. Kitt and colleagues (2025) used the same speaking and viewing task with a sample of adolescents with and without anxiety disorders. In contrast to Chen and colleagues (2021), they did not find a difference in dwell time between the two groups during the speaking task. However, their population was not only younger, but also a mixed anxiety group including specific phobias; and gaze avoidance as a safety behavior may be particularly relevant to SAD.

#### Disruptions while Speaking

Socially anxious individuals often have a hard time to keep speaking to an audience. This may be visible in speech disruptions, i.e., when the speaker temporarily stops conveying verbal information, and in speech duration, i.e., the total time a person spends speaking. Speech duration may be short when there are many disruptions, but also when a person quickly terminates the speech. For example, in a study on speech disruptions in socially anxious adults versus healthy controls, Hofmann et al. (1997) found that the SAD group had significantly more speech disruptions with both filled (e.g., “uhm”, “er”) and silent pauses. They explained the reason for these disruptions to be socially anxious individuals shifting their attentional resources to other cognitive processes, such as focusing on their own emotions and presence. Similarly, Levitan et al. (2012) found that adults with SAD were rated worse on intonation and speech fluency (absence of disruptions) than control participants. In an adolescent study, speech disruptions were observed in nearly a third of adolescents with high levels of social anxiety, compared to 20% in the low anxiety group (Blöte et al., 2015).

In summary, evidence to date points to a link between SAD and both eye-gaze avoidance and speech disruptions. Previous research has shown that anxious behavior of socially anxious adolescents during a public speaking task can have negative academic and social consequences including poorer grades (Blöte et al., 2007) and more negative treatment by class peers, including not listening attentively while the person is speaking and ridiculing the speaker (Blöte & Westenberg, 2007). Negative social experiences may exacerbate symptoms of SAD and cumulate to increased impairment (Asbrand et al., 2019; Rapee & Spence, 2004); reducing anxious behavior such as eye gaze avoidance and frequent speech disruptions via CBT could therefore help reverse the negative spiral.

### Changes in Anxiety Markers after Cognitive Behavior Therapy

Despite CBT being one of the best-documented forms of psychotherapy to treat anxious youth (James et al., 2020), there are scarcely any studies on the potential effects of CBT on specific behavioral markers of SAD. Apart from a study demonstrating positive effects of CBT on global observer ratings of socially anxious adolescents’ performance on behavioral assessment tasks (Herbert et al., 2009), we found only one prior study with an adolescent population, and three studies with adults on the effect of CBT on specific behaviors. These are summarized below.

Olivares-Olivares et al. (2019) studied the effect of treatment on socially anxious adolescents’ eye-contact during an interview; duration of eye contact was obtained by studying video recordings of the interviews. They found that participants who received group CBT, the Spanish *Intervention in Adolescents with Generalized Social Phobia* program, with an added focus on social skills training, maintained a significantly longer duration of eye contact with the experimenter than participants who received the same therapy without the social skills training. For both treatment groups, the improvement from pre-test to post test was significant, whereas a waitlist control group did not show a significant change in eye contact over the 12-week interval. As such, CBT, in particular when it incorporates social skills training, could potentially improve eye gaze during a public speaking task.

To the best of our knowledge, no studies have investigated the effect of CBT on speech disruptions among adolescent populations. In a randomized controlled trial (RCT), Beidel et al. (2014) studied total speech duration in socially anxious adults. They conducted an impromptu speech task before and after Social Effectiveness Therapy (SET) – a 12-week CBT program combining exposure tasks with social skills training. This was compared to a group with only exposure therapy, and a waitlist control group. They found SET led to superior improvements in total speech time, and in observer ratings of social skills.

Two RCTs offering only the exposure ingredient of CBT compared the effects on speech duration of in vivo exposure, virtual reality exposure and a waitlist control condition. Both studies recruited participants from the general public who met diagnostic criteria for SAD. Anderson and colleagues (2013) asked 97 participants to speak for ten minutes on up to three predetermined topics, before and after ten biweekly exposure sessions of 90 min. They did not find a significant difference between virtual reality exposure and in vivo exposure group therapy, or a difference between in vivo exposure group therapy and waitlist control. They did find a significant difference in post-test speech duration between virtual reality exposure and the waitlist condition. Kampmann and colleagues (2016) asked their 60 participants to perform a five-minute impromptu speech before and after eight exposure sessions. Speech duration improved significantly after both in vivo exposure and virtual reality exposure, compared to the waitlist control group. There was no difference in speech duration between the two exposure groups. Although the relative effectiveness varied between the three reviewed studies, together, they indicate treatment may have a positive effect on speech duration. Moreover, the lack of significant change in the waitlist control groups suggests that treatment, rather than the effect of time or exposure to the same task twice, is likely to be operational to the change.

### Effect of Age on Changes in Anxiety Markers after Cognitive Behavior Therapy

In the dynamic and continuously changing phase of adolescence it is relevant to investigate age as potential moderator of treatment’s influence on anxiety markers. Van den Bos and colleagues (2017) showed that stress responses change throughout adolescence and that pubertal developmental level is an important factor in neuroendocrine stress responses in adolescents. Leigh and Clark (2018) have suggested that avoidance safety behaviors may be more prevalent among younger adolescents whereas older adolescents may be more likely to use impression management. Avoidance safety behavior includes avoiding eye-contact, and an example of impression management would be to check how one is coming across (Evans et al., 2021). Public speaking behavior was related to age in a community sample of 9 to 16-year-olds: independent observer ratings of expressive and confident behaviors were positively related to age (Blöte et al., 2015). Furthermore, an increase in eye-contact during adolescence has been found in peer conversation studies. A study comparing children of different ages to adults found that adult male dyads made 40% more eye-contact than 10-year-old boys, while adult female dyads made 50% more eye-contact than 10-year-old girls (Levine & Sutton-Smith, 1973). Likewise, Van Beek et al. (2006) showed that 16-year-old girls made more eye-contact than 13-year-old girls. As relevant behaviors are still developing in adolescence, they may be more or less responsive to skills training in adolescents of different ages. Therefore, we include age as an exploratory moderator of a change in anxiety markers with treatment.

### The Present Study

In summary, to date there is a gap in empirical evidence investigating the potential influence of CBT on behavioral anxiety markers among socially anxious adolescents, despite the social benefits of improving these behaviors. We therefore aimed to study the extent to which CBT could alter socially anxious adolescents’ eye-gaze behavior and speech disruptions during a public speaking task, in addition to the traditionally reported indices of anxiety remission (Velthuizen et al., 2025). In line with the finding that disorder-specific treatment programs have higher effect sizes than generic ones (Reynolds et al., 2012) we chose a disorder-specific group CBT protocol designed specifically for socially anxious adolescents: the *Skills for Academic and Social Success* (SASS) program. This 12-week group intervention, developed in 2001 by Masia Warner and colleagues, is typically carried out in schools and, in addition to cognitive restructuring, focuses on practicing social skills and conducting social exposure tasks. Research has shown SASS to be an effective intervention, with medium to large treatment effects (*d* = 0.69 in Masia Warner et al., 2016; *d* = 0.76 in Miller et al., 2011) and remission rates of 50–67% (Masia et al., 2001; Masia Warner et al., 2005, 2007).

Before and after CBT, we administered a public speaking task with a subsequent viewing phase, as developed by Chen et al. (2021). Although other social interactions may be more frequent in the lives of adolescents, a public speaking context has the advantage that the audience does not have to make an active contribution to the interaction, so that the behavior of the socially anxious adolescent can be observed in a standardized situation. Adolescents are regularly asked to perform speeches at school in front of their class; our public speaking exercise was intended to capture and study their behavior in a naturalistic situation. We hypothesized that at post-treatment, participants would display more eye-gaze towards audience members’ faces (Olivares-Olivares et al., 2019), and longer speech duration (Anderson et al., 2013; Beidel et al., 2014; Kampmann et al., 2016) in the form of less disruption (Hofmann et al., 1997) compared to pre-treatment. Moreover, we explored whether changes after treatment were moderated by age. In addition, and in line with previous eye-tracking studies (Chen et al., 2021; Kitt et al., 2025) we used a task with both a speaking and viewing phase in order to establish whether gaze behavior changed in both phases.

## Method

### Participants

#### Recruitment

Adolescents aged 11–17 years were recruited from public secondary schools in and surrounding a moderately large Dutch city for participation in a treatment efficacy study (Velthuizen et al., 2025). All included participants had SAD as their primary concern, and reached a Clinical Severity Rating (CSR) score of at least four in the child or parent clinical interview (ADIS-C/P; Silverman et al., 2001). We excluded adolescents with diagnosed or strongly suspected autism spectrum disorder, adolescents with suicidal inclinations or other difficulties that required immediate attention, and adolescents with behavioral difficulties that would disrupt the group treatment. The study was approved by Leiden University’s Psychology Research Ethics Committee. All participants and their parents gave informed consent before therapy commenced.

#### Final Sample

**Fig. 1:**
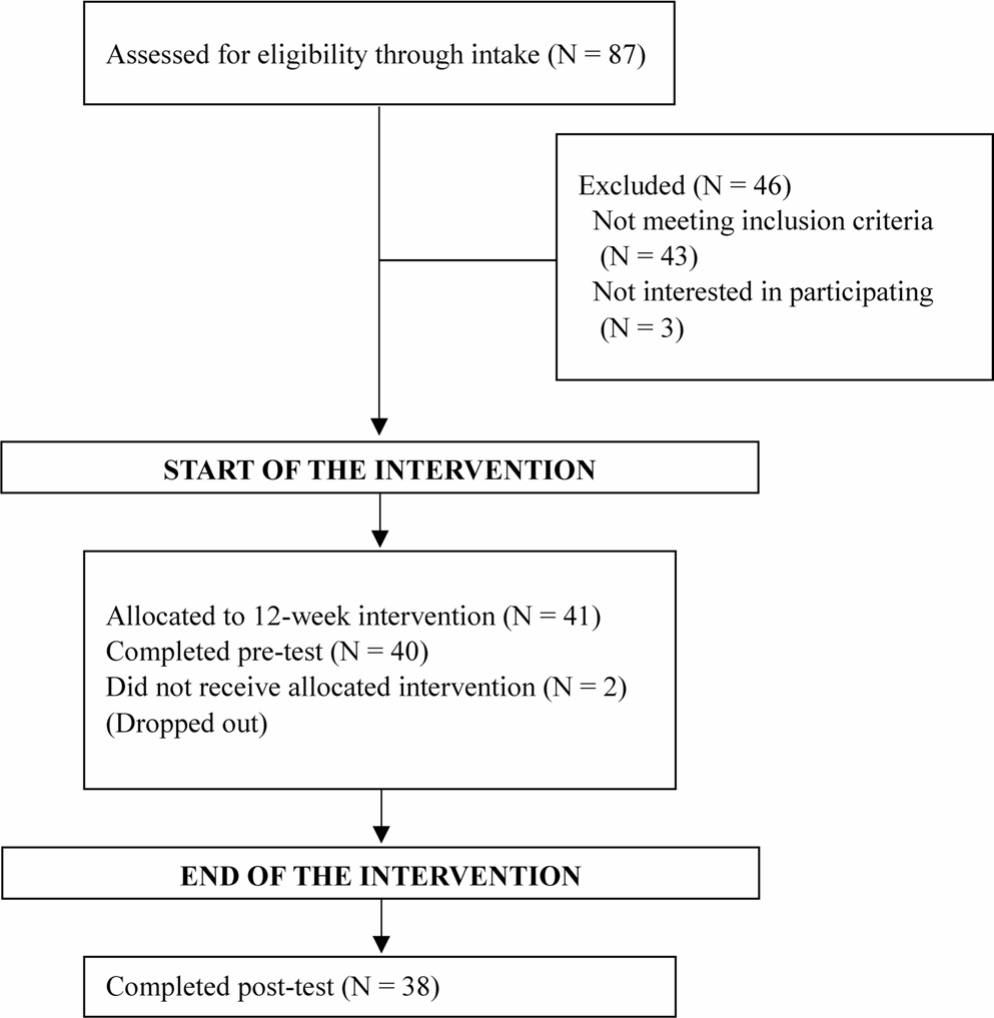
Flowchart of sample size at pre- and post-test data collection points

In total, 41 adolescents with a mean age of 14.46 (*SD* = 1.47) were recruited, see Fig. 1. The sample consisted of 20 girls (48.78%), 20 boys (48.78%) and 1 genderfluid participant (2.44%). Gender was assessed through the clinical interview with the adolescent; data on socioeconomic status and ethnicity were not collected. The sample as a whole was predominantly Caucasian, middle class, and from a moderately large Dutch city. The city has a slightly younger population with a higher educational level and income per inhabitant than the national average (AlleCijfers, 2025). Participants had a mean CSR-child score of 5.68 (SD = 1.21) at pre-test. The speaking task was completed by all participants except for one at T1 because the task was too demanding, and one at T2 because the participant was not available (speaking task *N* = 40 at T1; *N* = 38 at T2). In addition to the speaking task, all participants completed the viewing task, with the exception of the first therapy group because it was not yet introduced into the protocol (viewing task *N* = 33 at T1; *N* = 31 at T2). Two participants dropped out after the first therapy session.

### Study Design

These data were collected as part of a single-arm efficacy study, studying within-subject changes from before to after treatment (Velthuizen et al., 2025). Data collection took place at four time points: intake (T0), pre-test (T1), post-test (T2) and follow-up (T3). In the present study, we only use the data collected at pre-test (T1) and post-test (T2) which are the only two time points when the participants performed the public speaking task. These two time points took place in the week before the start of treatment and the week after the completion of the 12-week treatment. Data were collected by research assistants and graduate students. Therapists were occasionally involved in data collection at pre-test, but not at post-test. Researchers administering the public speaking task followed a protocol and practiced under supervision. Interviewers received extensive training in the clinical interview with regular supervision.

### Instruments

#### Eye-Tracking Glasses

To track eye gaze behavior during the public speaking exercise, wearable eye-tracking glasses, Tobii Pro Glasses 2 (Tobii AB, Sweden), were used. The experience is much like wearing reading or sunglasses, and is therefore not likely to evoke unrealistic distractions. The eye tracker is equipped with four eye cameras which track people’s eye movements in relation to the external environment they’re watching (field of view 90° 16:9, visual angle 82° horizontally and 52° vertically, resolution 1920 × 1080 pixels). It records eye gaze at a sampling frequency of 100 Hz and a scene video at 25 Hz. The speech audio was recorded by a microphone embedded in the eye-tracking glasses, allowing us to measure disruptions. The eye-tracker was controlled by Tobii Glasses controller software installed on a tablet through a wireless connection. The software was used for recording and calibration.

### Procedure

#### Treatment

The treatment consisted of 12 weekly group CBT sessions, *Skills for Academic and Social Success* (SASS; see Masia Warner et al., 2018 for the full protocol). Developed specifically for socially anxious adolescents, the protocol features cognitive restructuring and social exposure exercises in addition to social skills training. The protocol covers varying social situations, starting small with skills like initiating a conversation with your desk neighbor at school, and progresses through to more difficult tasks like making phone calls and public speaking tasks, to doing embarrassing things in front of strangers. As part of the program, the adolescents practice public speaking skills; this has the primary focus during one session but also recurs throughout in the form of short public speaking exercises in the group. Moreover, behaviors such as eye-contact, speaking volume, speech fluency, posture, and more, were practiced throughout the protocol through various social skill exercises. SASS was delivered in an outpatient university clinic which allowed us to offer sessions that are longer than a school lesson, increase standardization, and utilize more on-site expertise. Participants also had access to a supplemental blended element (mHealth) with a CBT-focus throughout the treatment period. Accessible via smartphone and laptop, the mHealth platform consisted of psychoeducation modules, relaxation exercises, cognitive restructuring practices, and more. It also had the possibility to chat with the therapy group as a whole, and to send direct messages to the therapists. Participants were given weekly homework assignments to complete on the mHealth platform, and were actively encouraged to use the platform beyond the homework assignments. Treatment outcome was not improved by greater mHealth usage (Velthuizen et al., 2025). Treatment groups consisted of age peers, with a maximum age difference of two years (e.g., 15–17-year-olds in one group). In total, nine treatment groups of 3–7 participants per group were facilitated. Treatment was led by two female therapists holding master’s degrees in child and adolescent psychology with clinical experience leading CBT-based treatment groups.

#### Public Speaking Task

The public speaking task was based on the Leiden Public Speaking Task (LPST; Westenberg et al., 2009) using the same preparation time, total speech duration and pre-recorded audience. The LPST was modeled on a classroom presentation, which Dutch students are regularly required to give in secondary school. It has previously been administered to 327 adolescents (van den Bos et al., 2017). In the present study, participants performed the public speaking task in front of the pre-recorded audience while wearing eye-tracking glasses at pre- and post-test. The audience consisted of eight age peers and one teacher, seated in a classroom. The audience showed neutral affect because this is more realistic than angry faces yet still evokes a threat-like response due to the social nature of a face (Schmidtendorf et al., 2022; Yoon & Zinbarg, 2008). Two recordings of age peers were used: one audience for 12–14-year-old participants (*N* = 15) and one for 15–17-year-old participants (*N* = 26). The audience was projected life-size in front of the participant. Participants were aware that the audience was a video recording, and not a live audience via videocall. Virtual audiences have been shown to be similarly stress-evoking as real audiences (Kothgassner et al., 2016) and these particular videorecorded audiences have been shown to evoke stress responses in adolescents (Kitt et al., 2025; van den Bos et al., 2017; Westenberg et al., 2009).

While the therapy had a group format, all data collection including the public speaking task was conducted individually in a research facility at the social science campus. The facility had a room for the public speaking task with a projector and large screen onto which the audience was projected life-size. The participant stayed in this room throughout the task. The researcher was in the room with the participant to give instructions, calibrate the eye-tracker and debrief the participant, but retreated into the adjacent control room when the participant prepared for the speech, delivered the speech and performed the viewing task. An intercom system allowed the researcher to communicate with the participant from the control room. Data was collected by the therapists (pre-test only), research assistants, and trained master’s students of psychology. The participants were first informed of the public speaking task during the intake; thus, it was not impromptu. Participants were instructed not to wear eye-makeup in case this interferes with the validity of the gaze data; makeup remover was available in the laboratory in case participants had overlooked these instructions.

The public speaking task consisted of one minute of introducing themselves, followed by four minutes of a speech on a particular topic. When introduced to the task, the participants were given examples of what to talk about when introducing themselves for the first minute (e.g., “You can talk about who you live with, where you go to school, what you like to do in your spare time, and such.”). This instruction was the same at both time points. For the main part of the speech, the participants were assigned a general topic to talk about (e.g., “Tell us about your favorite movie and why you like it.”); the topic was different at post-test. Here they were given examples of what types of things they can talk about: e.g., themes, actors, specific scenes, etc. They were asked to keep talking for four minutes. The participants were then given five minutes to mentally prepare their speech. They were not allowed to use any written material during their speech.

After the five minutes of preparation time, the participant put on the eye-tracking glasses, and these were calibrated using Tobii’s default one-point calibration followed by a four-point calibration check. Once calibrated, they were asked to stand on a specified spot at a distance of about two meters from the projector screen, which showed an empty classroom. After a short moment, the image showed a class entering the classroom and taking their seats. An auditory signal followed, at which the participant could start speaking. They introduced themselves and transitioned into their speech after they heard a second signal, a minute after the first signal. When a pause longer than 30s fell, they were prompted via the intercom system to try to continue: “Time is not up yet. Try to tell a bit more about the movie. Could you tell something more about…” with the experimenter suggesting an aspect of the topic they had not covered in detail yet. This prompt was carried out a maximum of three times. Participants were videotaped during the speaking task, which they were aware of.

Following the speaking task, the participants removed the eye-tracking glasses and completed a questionnaire about their perception of the audience. Once this was finished, they put on the eye-tracking glasses once again, these were calibrated following the same procedure, and then they stood before the audience, now with a clipboard and pen in hand. They were then asked to first simply look at the audience members for one minute, and to then rate each audience member in turn with the question “How would you rate the behavior of audience member number …?” on a 5-point Likert scale from positive to negative, as a number appeared above the audience member’s head on screen, similar to the procedure used by Chen et al. (2021). Data on audience perception are not reported here. Participants were debriefed after each public speaking measurement. Participants did not receive compensation.

### Data Preparation

#### Gaze Behavior

The eye-tracking data were processed using Tobii Pro Lab software. Pictures of the video being displayed on the screen were used as reference images, drawing areas of interest (AOIs) on the faces of the 9 audience members. In this study, the specific AOI lied within the outline of each audience member’s face, excluding the person’s hair. We used the Tobii I-VT (Velocity-Threshold Identification) Attention gaze filter, which has been designed for the use of eye-tracking glasses in dynamic situations. The attention filter identifies fixations using a velocity threshold of 100°/s and a minimum fixation duration of 60 milliseconds (ms). Adjacent fixations are merged when the time between fixations is no more than 75 ms and the distance between fixations is no more than 0.5°, based on the average data from both eyes (Olsen, 2012). The fixations that had been registered relative to the scene video were automatically mapped onto the reference images for the five minutes between the start and end signal of the speaking task and the one minute between the start and end signal of the viewing task. Subsequently, these mappings were checked by a human observer. The software calculated fixation-based parameters: the number of fixations on each AOI and the duration of fixations on each AOI in milliseconds. Fixation counts and fixation time were cumulated across AOIs and divided by duration of the task in minutes to obtain comparable data for the 5-minute speaking task and the 1-minute looking task.

The eye-tracker also coded each gaze sample as valid or invalid for each eye. Behaviors that would lead to the eye-tracker temporarily losing contact with the pupil include the participant blinking, closing their eyes, or moving their eyes in an extreme direction (e.g., keeping their head still but moving their gaze to their feet; turning their eyes up to the ceiling in thought). Raw data were exported from Tobii Pro Lab and imported in Matlab. A Matlab script was used to select the gaze samples pertaining to the 5-minute speaking task and the 1-minute looking task and compute the percentage of valid gaze samples (adding gaze samples for the left and right eye). The mean percentages of valid gaze samples were 84 (*SD* = 13) at T1 and 85 (*SD* = 12) at T2 for the speaking task and 90 (*SD* = 13) at T1 and 92 (*SD* = 8) at T2 for the viewing task. Seven data segments had less than 70% valid gaze samples. Since excluding these from the analyses did not affect the pattern of the results, the results reported are based on all available data.

#### Disruptions while Speaking

Although short silences and filled pauses commonly occur in spoken language, silences of more than two seconds have been shown to be rare in speech samples from a variety of contexts, including public speaking (Grosman et al., 2018). In the present study, participants were sometimes silent for a long time (Max = 54 s), suggesting a breakdown of speech performance. Likewise, some participants addressed the experimenter with a question or comment about the task (e.g., “How much time is left?”, “I don’t know what to say anymore”). Therefore, silences and filled pauses (i.e., silences interspersed with utterances that don’t contribute to the meaning of the sentence such as, “uhm”, “right”) of more than two seconds and interactions with the experimenter were manually coded as disruptions in Tobii Pro Lab, by marking their start and end times in the recording. After an exploratory phase of listening to the recordings and making notes about conspicuous disruptions, the following procedure for data coding was established and applied to all recordings by the same coder. The coder listened to the recording and placed a marker at the end of a disruption. Then the coder listened to the preceding part of the recording again to mark the beginning of the disruption. If the beginning and end points were more than two seconds apart, the markers were kept; otherwise, they were deleted. Subsequently, the number and duration of disruptions were totaled and divided by the total duration of the speech to obtain the number of disruptions per minute and the duration of disruptions in milliseconds per minute.

### Statistical Analysis

We analyzed the differences in gaze behavior and speech disruptions between pre- and post-test, and moderation by age. Gaze behavior and speech disruptions were both measured in number and in duration. Age was used as a continuous variable. In total there were four dependent variables: number of fixations, duration of fixations, number of disruptions and duration of disruptions. The four dependent variables were analyzed using linear mixed models with task (for gaze behavior only), age, and interactions of time point with each of the variables as predictors. Significant effects of task were followed up by separate analyses per task condition (speaking and viewing). Effect sizes were interpreted using Cohen’s (1988) d standards (small = 0.2, medium = 0.5, and large = 0.8). Analyses were conducted in RStudio version 4.3.1 using base packages (R Core Team, 2013), effsize package (version 0.8.1; Torchiano, 2020), the lme4 package (version 1.1–35.3; Bates et al., 2015), the dplyr package (version 1.1.3; Wickham et al., 2023) and the ggplot2 package (version 4.3.3; Wickham, 2016).

## Results

See Table 1 for the means and standard deviations of the total sample, and Table 2 for an overview of the main results. The dependent variables included in the analyses were square root transformed to better approximate the normal distribution. The two eye-tracking variables were highly correlated (*r* =.88, *p* <.001), as were the two disruptions variables (*r* =.79, *p* =.001).

**Table 1:** Means and standard deviations

	*T1*	*T2*
	M (SD)	M (SD)
**Eye-gaze**		
*Number of fixations*		
Speaking	9.07 (6.53)	11.8 (9.85)
Viewing	18.5 (10.5)	19.0 (15.4)
*Duration of fixations*		
Speaking	3.91 (2.89)	4.85 (4.15)
Viewing	11.89 (7.21)	12.39 (9.83)
**Disruptions while speaking**		
*Number of disruptions*		
	1.26 (0.75)	1.19 (0.77)
*Duration of disruptions*		
	10.37 (7.34)	10.37 (8.22)

Reported data are untransformed; duration reported in seconds

**Table 2:** Effect of treatment from pre- to post-test

**Interaction**	***Estimate***	***S.E.***	***df***	***t-value***	***p***
**Eye-Gaze**					
*Number of fixations*					
Time point	-4.85	1.79	97.66	-2.71	0.008 **
Age	-0.10	0.12	62.72	-0.81	0.42
Time point: Age	0.36	0.12	97.45	2.96	0.004 **
Task viewing	1.29	0.26	99.38	4.90	< 0.001 ***
Time point: Task viewing	-0.44	0.37	95.95	-1.20	0.233
*Duration of fixations*					
Time point	-106.94	46.91	98.58	-2.28	0.025 *
Age	-3.12	2.97	68.30	-1.05	0.297
Time point: Age	7.83	3.21	98.36	2.44	0.016 *
Task viewing	43.80	6.88	100.50	6.37	< 0.001 ***
Time point: Task viewing	-4.87	9.72	96.69	-0.50	0.618

### Eye Gaze

#### Number of Fixations on Faces

**Fig. 2:**
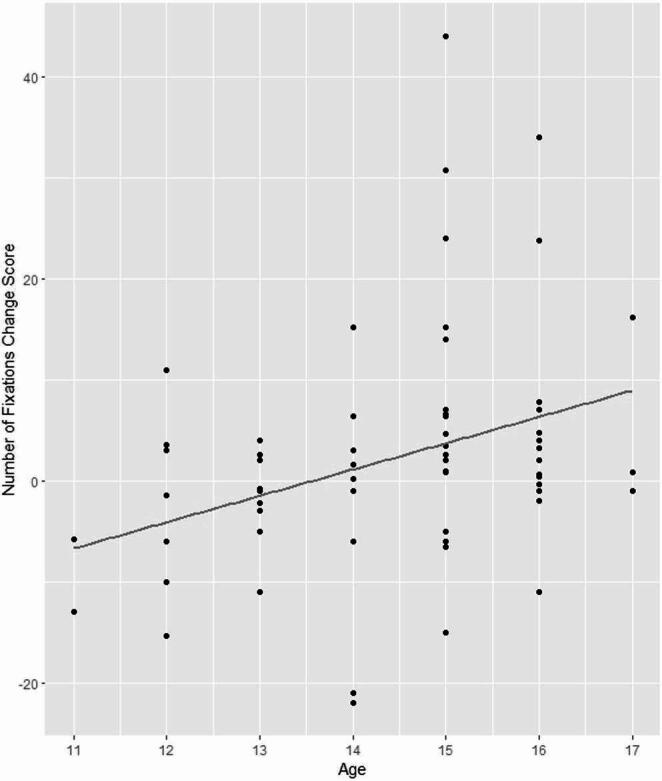
Age versus change score of number of fixations on audience members’ faces, showing greater change among older adolescents

The number of fixations on audience members’ faces showed a significant main effect of time with a large effect size (*t*(97.66) = −2.71, *p* =.008; *Cohen’s d* = −2.87, 95% CI [−3.18, −2.56]), qualified by a significant interaction with age with a small effect size (*t*(97.45) = 2.96, *p* =.004; *d* = 0.22, 95% CI [−0.00, 0.43]). Older participants showed a larger increase in the number of fixations on faces, see Fig. 2. The main effect of task was significant with a large effect size (*t*(99.38) = 4.90, *p* <.001; *d* = −0.75, 95% CI [−0.97, −0.54]). While the interaction between task and time was not significant t(95.95) = −1.20, *ns*), its effect size was medium (*d* = −0.44, 95% CI [−0.66, −0.22]), warranting follow-up analyses of the interaction between task and time. Separate analyses by task showed that there was a significant increase in number of fixations between pre-test and post-test during the speaking task (*t*(37.71) = 2.10, *p* =.042) with a small effect size, *d* = 0.31, 95% CI [−0.14, 0.77]; there was no significant interaction during the viewing task (*t*(30.76) = −0.53, *ns*).

#### Duration of Fixations on Faces

**Fig. 3:**
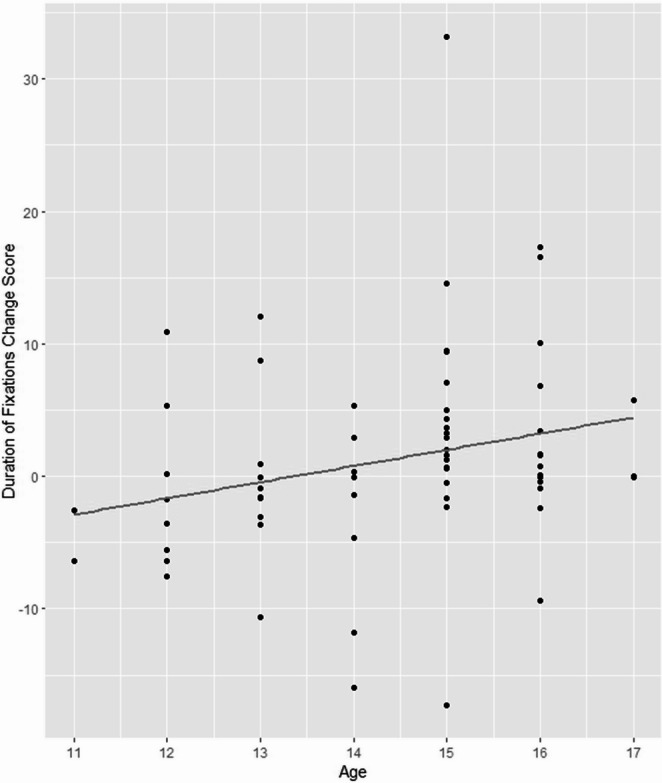
Age versus change score of duration of fixation on audience members’ faces, showing greater change among older adolescents

Similar to the number of fixations, duration of fixations on audience members’ faces showed a significant main effect of time with a large effect size (*t*(98.58) = −2.28, *p* =.025; *d* = −2.70, 95% CI [−3.00, −2.40]), qualified by a significant interaction with age (*t*(98.36) = 2.44, *p* =.016; *d* = 0.20, 95% CI [−0.02, 0.41]). Older participants showed a larger increase in the duration of fixations on faces, see Fig. 3. The main effect of task was significant with a large effect size (*t*(100.50) = 6.37, *p* <.001; *d* = −1.21, 95% CI [−1.55, −0.88]); however, the interaction between time and task was not significant and had a small effect size (*t*(96.69) = −0.50, *ns*; *d* = −0.12, 95% CI [−0.34, 0.09]).

### Disruptions while Speaking

There was no effect of time on number of disruptions while speaking (*t*(36.45) = 1.81, *ns*). Age was not a moderating variable (*t*(36.36) = −1.87, *ns*). Similarly, duration of disruptions also did not show an effect of time (*t*(34.54) = 1.01, *ns*), and no moderating effect of age (*t*(34.49) = −1.09, *ns*).

## Discussion

The present study investigated the extent to which disorder-specific group CBT for socially anxious adolescents improved behavioral markers of anxiety, specifically eye-gaze and speech disruptions, during public speaking. We also tested whether changes were moderated by age. Participants completed a public speaking and viewing task while wearing an eye-tracker, before and after 12 weeks of CBT. We found that subsequent to treatment, participants displayed a greater degree of eye-gaze towards the audience’s faces, both in number and in duration of fixations. The change was greater in older participants. We did not observe a difference in speech disruptions after CBT. Due to the small sample size of the present study, findings should be viewed as preliminary.

The significant increase in eye-gaze at post-test could indicate an influence of treatment. These results are in line with the only other adolescent study on the effects of CBT on these specific anxiety markers; Olivares-Olivares et al. (2019) found that CBT can have an effect on eye-contact during an interview, and that social skills training seems to be a particular driver of this effect. Age moderated the change of gaze behavior, indicating that gaze behavior improved more in older participants. Previous developmental studies demonstrated that older adolescents showed more expressive and confident behavior during public speaking than younger ones (Blöte et al., 2015) and that 16-year-old girls made more eye-contact during a conversation than 13-year-olds (Van Beek et al., 2006). Leigh and Clark (2018) suggested that avoidance safety behaviors, such as avoidance of eye-contact, may be more commonly observed among younger adolescents whereas older adolescents are more likely to use impression management strategies. This suggests that older adolescents may be developmentally more responsive to fine-tuning these social behaviors and to reduce avoidance safety behavior.

In the present study, an increase in number of fixations following CBT was observed in the speaking task but not during the viewing task. A change occurring in the speaking task only could support the notion that socially anxious individuals avoid eye-contact in socially evaluative situations, such as a speech, but not during non-threatening situations such as simply looking at an audience (Chen et al., 2021). However, given that the interaction between time and task was not significant, we cannot rule out that gaze behavior changed in the viewing task as well. Further research with a larger sample size is needed to clarify whether adolescents with SAD, like adults (Chen et al., 2020), avoid looking at faces in non-evaluative situations.

In line with two other studies comparing speaking and viewing (Chen et al., 2021; Kitt et al., 2025), participants looked more frequently and for longer at faces during the viewing task than during the speaking task. The mean fixation duration during the viewing task (see Table 1) was also comparable to the results of Chen et al. (2021), obtained with a community sample of young adult females (9.3–10.5 s), and the results of Kitt et al. (2025), obtained with an adolescent sample consisting of participants with an anxiety disorder and control participants (12.97s). However, the mean fixation duration during the speaking task was lower in the present study (Chen et al., 2021: 6.5–10.1 s; Kitt et al., 2025: 6.08s). This might be related to our participants being exclusively socially anxious adolescents as opposed to an adult community sample or a mixed anxiety population. However, there was no significant relation with social anxiety symptoms in Kitt et al.’s study (2025). Rather, the difference may be related to the duration of the speaking task: four minutes in the present study versus one minute in the other two studies. Our more demanding task poses a higher risk of running out of things to say. During disruptions, participants tended to look down or turn towards the control room when addressing the experimenter. Hence, lower mean numbers and durations of fixations on the audience may be representative of a longer speech task.

While different behavioral factors involved in giving a speech were practiced during the treatment, this practice did not seem to have a substantial effect on speech disruptions. This was contrary to our expectations and previous treatment studies among adult samples (Anderson et al., 2013; Beidel et al., 2014; Kampmann et al., 2016). In these studies, participants were allowed to terminate their speech after a minimum duration (Beidel et al., 2014: 3 out of 10 min) or when they wanted to stop (Kampmann et al., 2016). In the present study, participants were encouraged to continue if they did not speak for the full five minutes and this may have obscured the effect. The change in eye-gaze behavior but not in speech disruptions could suggest eye-contact was given comparatively more attention during treatment, and in a greater variety of formats: when practicing public speaking but also when practicing one-to-one conversations, listening skills, assertiveness, etc. Another possibility may be that pausing is more automatic compared to shifting eye-gaze away from the other and therefore harder to correct. Although previous studies showed an increase in total speech duration measured from the start to the end of the speech (Anderson et al., 2013; Beidel et al., 2014; Kampmann et al., 2016), this does not necessarily imply a reduction of disruptions. More research is needed to clarify effects of CBT on different aspects of speech behavior, also including volume, speed, and intonation, that were practiced, but could not be addressed within the scope of this study.

Overall, due to a lack of comparison to healthy controls, we cannot draw conclusions as to how typical the speech disruptions were at either time point. Moreover, generalization of cognitive restructuring and social skills learned during CBT to actual social situations could take more time than our study allowed. The scope of this study covered the time points immediately before and after 12 weeks of CBT. However, as consistently observed in previous research using *SASS*, additional improvements are often made in the period following treatment (Masia Warner et al., 2007). Thus, the inclusion of follow-up measurements using a public speaking task should be considered, as additional benefits in behavior may evolve with more time. There were no relations between age and speech disruptions. Effects of age need to be studied in greater detail as there may be nuanced differences in safety behaviors (Leigh & Clark, 2018), not captured here.

### Clinical Implications

Specific aspects of social skills, including public speaking, can have an influence on adolescents’ school and peer functioning (Blöte et al., 2007; Blöte & Westenberg, 2007). The finding of a significant improvement in gaze behavior during public speaking after CBT has clinical relevance, as increased eye gaze can potentially reverse the negative spiral by reducing negative responses from others, improving social interactions and consequently leading to a reduction in impairment. The inclusion of public speaking training in treatment for socially anxious adolescents is a significant driver of symptom reduction (García-López et al., 2002). While public speaking skills were a recurring theme within our chosen treatment program, there are numerous other themes and skills covered throughout the twelve weeks of SASS. Thus, to see greater change in speech duration, more specific public speaking training within CBT may be needed. It has been suggested that, generally, more emphasis may need to be placed on positive peer feedback in the group setting (Miers & Masia Warner, 2023). In addition, Blöte et al. (2019) suggested socially anxious adolescents who were poor performers at public speaking would benefit from specific social skill training paired with cognitive restructuring, whereas socially anxious adolescents with high quality performances may benefit more from video-feedback interventions. Future research could test the influence of video feedback and structured positive peer response on behavioral outcomes.

### Limitations and Directions for Future Research

This study extends current literature by studying changes in eye-gaze over the course of CBT using eye-tracking technology, thus combining methodological strengths from previous research that has either studied the effect of CBT (Olivares-Olivares et al., 2019) or used precise eye-tracking technology but only at one time point (Kitt et al., 2025). Moreover, the use of the material and procedure from the Leiden Public Speaking Task (Westenberg et al., 2009) enabled the comparison of results across other studies that have used the same format (e.g., Chen et al., 2021; Kitt et al., 2025).

While this study provides novel insights, it also has a number of limitations which could be addressed in future research. In addition to replication with a larger sample to increase the power to detect small effect sizes, a primary recommendation for future research is to include a control condition. This could be done in two ways: first, inclusion of a non-anxious control group to delineate typical public speaking behavior from what is unique to socially anxious adolescents (see Kitt et al., 2025). While we observed an improvement in number of fixations in the speaking task, we did not observe an effect in the viewing task. Previous studies have suggested that socially anxious individuals show similar gaze patterns to non-anxious individuals during tasks where they are not judged (Chen et al., 2020); however, as our study did not include a non-anxious control group, we cannot be certain to what extent participants’ gaze behavior was on par with age peers. Second, use of an active control or a waitlist control condition, employed within a randomized design and with a larger sample size, would be necessary to draw stronger conclusions about the observed effects of CBT rather than potential habituation to the public speaking task. However, previous studies using a waitlist control with five weeks (Anderson et al., 2013), eight weeks (Kampmann et al., 2016) and 12 weeks (Olivares-Olivares et al., 2019) between measurement points did not see significant changes within their control group. Thereby, indicating that habituation of task may not be the prime operator behind the change observed in our twelve-week intervention period.

Moreover, this study utilized a video of an audience and not a live audience. This has the advantage of allowing for standardization but the disadvantage of lower ecological validity. A strength is that our audience was comprised of ‘real’ people rather than computer-animated people. However, it was nonetheless a video and the participants were aware of this; for example, there was no deception involved to suggest it was a videocall. Evidence shows that public speaking tasks elicit a stress response in adolescents (Miers et al., 2011; Westenberg et al., 2009) and it was observed to be demanding for many of our participants. It is not clear to what extent our participants would have behaved differently in front of a live audience. Future research should investigate the effects of treatment on behavioral markers in front of a live audience or during peer interaction. Such approaches would reduce standardization but may improve validity and generalizability of results.

There are numerous potential behavioral markers of SAD which may be present during a public speaking task, but which were not captured in this research (e.g., posture, facial expressions, volume). Future studies should code for other improvements in speech, both specific and global, following treatment using a coding scheme such as the Speech Performance Observation Scale for Youth (Blöte et al., 2015). Moreover, pubertal developmental level has been shown to be important in relation to (neuroendocrine) stress responses in this population (van den Bos et al., 2017); future studies should include this factor in addition to age. Previous studies have also reported gender differences in behavioral anxiety markers (Blöte et al., 2015; Levine & Sutton-Smith, 1973; Van Beek et al., 2006). The current study did not have sufficient power to study the effect of gender in a meaningful way.Future studies with larger sample sizes should consider studying whether gender influences the effect of treatment on these markers. Furthermore, future studies may test the effect of CBT on more social interaction skills beyond public speaking.

Lastly, we did not include a measure of state anxiety which would have been a relevant variable to capture in relation to behavioral measures, for example to provide insight into a potential mechanism of change for eye gaze and to rule out that low state anxiety contributed to the lack of change in speech disruptions. Future research into socially anxious adolescents’ behavior during public speaking tasks could include state anxiety measures such as a visual analog scale (Abend et al., 2014).

## Conclusion

The current study explored, for the first time, the potential effect of CBT for socially anxious adolescents on specific behavioral markers of anxiety during a public speaking task. Our findings reflect the notion that socially anxious individuals avoid eye-gaze in socially evaluative situations. Moreover, our results support that gaze behavior may be altered through general CBT treatment. The study also highlights the importance of including moderators such as age and possibly gender in adolescent treatment studies. Whilst replication is required, the behavioral improvement in eye gaze demonstrates the potential for bringing about more rewarding social situations – particularly with same-age peers – thereby yielding social gains for this socially vulnerable adolescent population. Future research should include randomized control groups for a greater understanding of adolescents’ typical behavior during public speaking tasks and to investigate the effect of treatment-related behavioral changes.

## Data Availability

Data can be made available upon request.

## Abbreviations

SAD: Social Anxiety Disorder
CBT: Cognitive Behavior Therapy
SASS: Skills for Academic and Social Success
